# Antifungal Activity of Menthol, Eugenol and Their Combination against *Aspergillus ochraceus* and *Aspergillus niger* In Vitro and in Stored Cereals

**DOI:** 10.3390/foods12112108

**Published:** 2023-05-24

**Authors:** Yamina Ben Miri, Ahmed Nouasri, Marta Herrera, Djamel Djenane, Agustín Ariño

**Affiliations:** 1 Department of Biochemistry and Microbiology, Faculty of Sciences, Mohamed Boudiaf University, P.O. Box 166, M’sila 28000, Algeria; sys_yamina@yahoo.com; 2 Food Quality and Safety Research Laboratory, Department of Food Sciences, Mouloud Mammeri University, P.O. Box 17, Tizi-Ouzou 15000, Algeria; 3 Laboratory of Bioactive Products and Biomass Valorization Research, ENS Kouba, P.O. Box 92, Vieux-Kouba, Algiers 16308, Algeria; a_nouasridz2001@yahoo.fr; 4 Facultad de Veterinaria, Instituto Agroalimentario de Aragón-IA2, Universidad de Zaragoza-CITA, 50013 Zaragoza, Spain; herremar@unizar.es (M.H.); aarino@unizar.es (A.A.)

**Keywords:** menthol, eugenol, synergistic effect, antifungal, cereals

## Abstract

*Aspergillus ochraceus* and *Aspergillus niger* are spoilage and mycotoxin-producing fungi that can contaminate agricultural commodities and derived products. In the present study, menthol, eugenol, and their combination (mix 1:1) were tested to determine their contact and fumigation toxicity against the two fungi. Menthol, eugenol, and their mixture significantly reduced mycelial growth and spore germination at concentrations from 300 to 600 µg/mL, and the inhibitory effects showed clear dose dependence. The minimum inhibitory concentration (MIC) values against *A. ochraceus* were 500 µg/mL (menthol), 400 µg/mL (eugenol), and 300 µg/mL (mix 1:1), while the MIC values for *A. niger* were 500 µg/mL (menthol), 600 µg/mL (eugenol), and 400 µg/mL (mix 1:1). Additionally, the analyzed compounds exhibited more than 50% protection against *A. ochraceus* and *A. niger* by fumigation of stored cereal grains (maize, barley, and rice) in sealed containers. The binary mixture of menthol and eugenol showed synergistic effects against both fungi in both in vitro direct contact and stored grain fumigation trials. The results of the present study provide a scientific basis for the application of a combination of natural antifungals in food preservation.

## 1. Introduction

Filamentous fungi, which are abundant in diverse habitats, not only contaminate our air, but also our food. As the mold grows on food, they produce enzymes that break down the food, resulting in spoilage. Food spoilage due to mold includes off-flavors, off-aromas, discoloration, and rotting, and can occur pre- and post-harvest [1]. Mold spoilage can also be a food safety issue due to the production of mycotoxins, as the ingestion of mycotoxin-contaminated food poses a significant health risk [2]. The mold species *Aspergillus ochraceus* and *Aspergillus niger* are commonly found on agricultural commodities and their derived products, both at the pre- and post-harvest stages [3]. Both fungi species are important as food spoilage molds, but they are also capable of synthesizing the mycotoxin ochratoxin A (OTA), especially *A. ochraceus* and less frequently *A. niger* [4]. The International Agency for Research on Cancer (IARC) has classified OTA in group 2B as possibly carcinogenic to humans. It is well known that the consumption of cereals and cereal products contributes significantly to the intake of ochratoxin by the population [5].

Proper use of chemical fungicides could reduce fungal contamination in food and feed; however, awareness on the toxicity of chemical treatments, pest resurgence and resistance, and environmental pollution has prompted research towards green, safe, and non-toxic applications in fungal control [6]. Essential oils (EOs) or their constituents have been suggested as a natural source of alternative fungicides for cereals, grains, pulses, fruits, and vegetables [7]. Some EOs extracted from aromatic plants have been shown to have fungicidal effects on *Aspergillus* molds colonizing food [8], and a remarkable ability to reduce mycotoxin synthesis [9]. The applications of EOs and their constituents as natural antifungals have been extensively studied in wheat grains [10], bread products [11], and bakery products [12].

The biological activity of EOs is attributed to the presence of major constituents such as bioactive terpenes and phenolic aromatic molecules [13]. Menthol is a volatile cyclic monoterpene alcohol with remarkable biological properties that is used in a variety of foods and medicinal products [14]. This compound is found as a major constituent in the essential oils of several plants of the *Mentha* genus in the *Lamiaceae* family. Menthol is known to exhibit biological activity in vitro and in vivo, and is an effective fumigant [15]. On the other hand, eugenol is a volatile phenolic monoterpenoid, which is the foremost constituent in clove essential oil (*Syzygium aromaticum*). Eugenol is generally derived from plant essential oils of several families, such as *Myrtaceae*, *Lauraceae*, and others [16]. The possible synergistic effect produced by the combination of different compounds is an efficient strategy for inhibiting microbial development, and reducing individual concentrations of active agents and possible adverse sensory effects for food application.

The antifungal activity of menthol and eugenol, as well as their possible synergistic effects on common foodborne fungi, needs consideration. Screening for bioactivity is commonly performed in vitro in bioassays for antifungal activity with contact methods using agar or liquid media. Additionally, the vapor toxicity should be studied in a food model to simulate fumigation during storage. Fumigation is one of the best methods to prevent the fungal contamination during storage with no lingering or no/little residual effect. Thus, the objectives of the present study were to investigate the contact toxicity of menthol, eugenol, and their combination on the fungal growth and spore germination of *A. ochraceus* and *A. niger*, as well as to evaluate their efficacy as natural fumigants during storage of cereal grains.

## 2. Materials and Methods

### 2.1. Chemicals, Solvents and Culture Media

Menthol (purity 99%) and eugenol (purity 98%) were purchased from Sigma-Aldrich France (Merck KGaA, Darmstadt, Germany) and stored at +4 °C in the dark until use. Tween 80 and HPLC grade solvent dimethyl sulfoxide were provided by Sigma-Aldrich France. Potato Dextrose Agar (PDA), Potato Dextrose Broth (PDB) and Dichloran Rose Bengale Chloramphenicol (DRBC) agar were purchased from Merck KgaA (Darmstadt, Germany).

### 2.2. Fungal Strains

Strains of *A. ochraceus* and *A. niger* were kindly provided by the Microbial Systems Biology Laboratory (LBSM) of the School Normal Superior of Kouba in Algiers (Algeria), and both strains were isolated from Algerian maize. Spore inoculum of each fungus was prepared from 7-day-old cultures on PDA at 25 °C by washing the Petri dish with 10 mL sterile distilled water containing 0.1% (*v*/*v*) Tween-80 and rubbing the surface with a sterile glass rod. The conidial suspension was adjusted to 10^6^ spores/mL by counting with a hemocytometer slide (depth 0.2 mm, 1/400 mm^2^) under a light microscope (Motic BA210, Xiamen, China).

### 2.3. In Vitro Activity on Mycelial Growth

The evaluation of the activity of menthol, eugenol, and their combination (mix 1:1) on the mycelial growth of *A. ochraceus* and *A. niger* was carried out using the direct contact method on PDA plates [17]. Stock solutions of antifungals were prepared in 1% dimethyl sulfoxide (DMSO). The solutions of test compounds were incorporated in an autoclaved PDA medium cooled to 55 °C to achieve final concentrations of a 100, 200, 300, 400, 500, and 600 µg/mL PDA medium (equivalent to 0.01–0.06%). The binary mixture 1:1 (50% each) was made by adding equal parts menthol and eugenol. After solidification, plates were inoculated with 10 μL of the conidial suspension deposited in the center of the culture medium. A control treatment, containing only the DMSO solution and the culture medium, was also employed.

Plates were incubated at 25 °C and examined at 7 days for colony diameter measurements. Two diameters at right angles were measured for each colony. Percent inhibition at each antifungal concentration was calculated from the mean colony diameter of the control plates and the mean colony diameter on each fungicide-amended plate. A linear regression of colony radial growth rate versus fungicide concentration was calculated for each isolate, and the effective fungicide concentration (EC) estimated to produce 50% inhibition (EC_50_) and 90% inhibition (EC_90_) were determined by interpolation from the regression line [17].

Additionally, the minimum inhibitory (MIC) and fungicidal (MFC) concentrations were determined using the liquid dilution method [18] in Potato Dextrose Broth (PDB). Ten (10) µL of the conidial suspension were inoculated into test tubes of 10 mL of PDB containing the antifungal agents at different concentrations (100 to 600 µg/mL). PDB tubes containing DMSO solution were used as the control. The tubes were homogenized and incubated at 25 °C for 7 days. The MIC value corresponded to the lowest concentration that inhibited the growth of test molds. Tubes that showed no visible growth were subcultured on PDA plates to determine the MFCs. MFC is the lowest concentration of antifungal at which there was no revival of growth of the inhibited fungal inoculum because of permanent inhibition.

### 2.4. Spore Germination Assay

An aliquot of 100 μL of conidial suspension containing 10^5^ spores was incubated with 100 μL of a DMSO solution of menthol, eugenol, or their mixture (1:1) in the concentrations of 100 to 600 µg/mL using sterile glass depression slides [19]. The depression slides were incubated at 25 °C for 24 h in a Petri dish containing wet filter paper. A control treatment, containing only the DMSO solution and the inoculum, was also employed. Inhibition of germination was observed under light microscopy and the spore germination percentage was calculated by counting the number of germinated spores for a total 100 spores. A spore was considered germinated when the length of the germination tube was longer than the diameter of the spore [17].

### 2.5. Fumigation in Stored Cereal Grains

To determine the fumigant toxicity of menthol, eugenol, and their combination (mix 1:1) against *A. ochraceus* and *A. niger* in cereal grains, a bioassay method at laboratory scale was used as previously described [10]. Grains of maize, barley, and rice were procured from a local market in Reghaïa (Algeria), placed in sterile bags, and brought to the laboratory. The cereal grains were surface-sterilized with a 1% solution of sodium hypochlorite, rinsed 3 times with sterilized distilled water, and dried overnight in a laminar flow hood (Telstar, Japan). The antifungal efficacy of menthol, eugenol, and their combination (mix 1:1) was estimated by separately storing 1 kg of each sample for six months at room temperature in 2 L airtight plastic containers. One milliliter (1 mL containing 10^6^ spores) of conidial suspension of *A. ochraceus* or *A. niger* were uniformly sprayed on the cereal grains. For this purpose, 100 µL aliquots of spore suspension of each isolate were inoculated at 10 different points on the grain mass, which was then thoroughly homogenized until the final fungal load of 10^3^ fungal spores per gram of cereal was reached. Then, menthol, eugenol, and their combination were applied on sterile cotton swabs attached to the inner-surface of the container caps, and impregnated at their MIC values with respect to the aerial volume of the container. For the calculations, MIC values in the agar medium (µg fungicide/mL agar) were converted into vapor-phase concentrations in air volume (µg fungicide/mL air) to give final concentrations between 400 and 600 µg/mL of air. The containers including the inoculated cereal grains and the antifungals were tightly closed and further sealed with parafilm. Similar sets without test compounds were arranged and served as the control.

For mycological analysis, the spread-plate method was used. Ten grams of treated and control grain samples was placed in 250 mL flasks containing 90 mL of sterile Tween water (0.1%) and blended for 1 min. Serial decimal dilutions up to 10^−3^ were made, 0.1 mL of each dilution was inoculated on DRBC agar plates, and the inoculum was spread with a sterile, bent glass rod. Duplicate plates were incubated in the dark at 25 °C for 5 days, and the count was expressed as cfu/g. The percent protection (%P) of cereals was calculated based on the plate counts of *A. ochraceus*/*A. niger* in treatment and control samples as follows:

(1) $$\%P=\frac{\mathrm{Fc}-\mathrm{Ft}}{\mathrm{Fc}}\times 100$$

%P = percent protection; Fc = counts of *A. ochraceus*/*A. niger* (cfu/g) from control samples; Ft = counts of *A. ochraceus*/*A. niger* (cfu/g) from treated samples.

### 2.6. Statistical Analysis

The results were statistically analyzed using one-way ANOVA and Tukey’s post hoc test (STATISTICA 6.0, StatSoft Inc., Tulsa, OK, USA). Results with *p* < 0.05 were considered statistically significant.

## 3. Results and Discussion

### Effects of Natural Antifungals on Mycelial Growth

**Figure 1:**
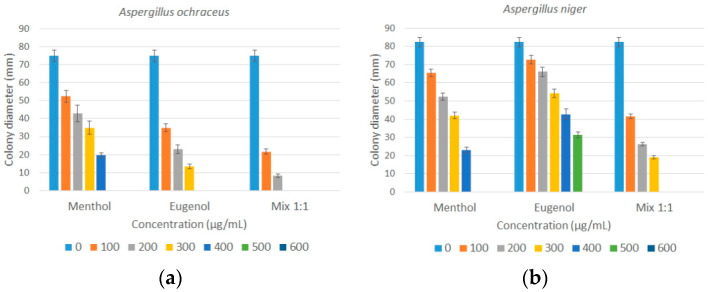
Effects of menthol, eugenol, and their combination (mix 1:1) on the growth: (**a**) *A. ochraceus*; (**b**) *A. niger*. Colony diameter (mm) expressed as mean ± standard deviation of three replicates.

The effects of menthol, eugenol, and their combination (mix 1:1) on the growth of *A. ochraceus* and *A. niger* during the seven days of incubation are presented in Figure 1. The growth of mycelium (colony diameter) of both molds was significantly reduced (*p* < 0.05) in a proportional way to the concentrations of the antifungals, indicating dose-dependent activity. *A. ochraceus* was more sensitive to eugenol than to menthol, but it was most sensitive to a mixture of both agents. *A. niger*, on the other hand, was more sensitive to menthol than to eugenol, although it was also more sensitive to a binary mixture of menthol and eugenol (mix 1:1).

Menthol reduced the mycelial growth of *A. ochraceus* at concentrations of 100, 200, 300, and 400 µg/mL with reduction percentages of 30.2%, 42.8%, 52.9%, and 73.8%, respectively, while total inhibition (100% reduction) was obtained at 500 µg/mL. Percent inhibition of *A. ochraceus* growth with eugenol ranged from 53.3 to 82.2% when exposed to levels of 100 to 300 µg/mL, and complete inhibition was achieved at 400 µg/mL and beyond. However, the menthol-eugenol binary mixture (1:1) showed a synergistic effect because at only 100 and 200 µg/mL, the inhibition was 71.1% and 88.9%, respectively, while from 300 µg/mL, the growth of *A. ochraceus* was totally inhibited (Figure 1a). The synergism is achieved when the combination of antifungals has a higher inhibitory effect than the inhibitory activities of individual compounds.

On the other hand, menthol showed similar antifungal activity against *A. niger*, ranging from a 20.0 to 71.9% reduction of mycelial growth at 100–400 µg/mL concentrations, and total inhibition from 500 µg/mL. The antifungal activity of eugenol at concentrations between 100 and 500 µg/mL caused an 11.7 to 61.9% reduction in mycelial growth, with complete inhibition at only 600 µg/mL. Likewise, the combination menthol-eugenol (mix 1:1) showed a synergistic effect against *A. niger*, as mycelial reduction attained 49.4 to 76.9% at levels of 100–300 µg/mL, and the fungus was completely inhibited at 400 µg/mL (Figure 1b).

The mean concentration of fungicides that inhibited the size of colonies of *A. ochraceus* and *A. niger* on PDA by 50% (EC_50_) and 90% (EC_90_) at 7 days of incubation are shown in Table 1. These EC_50_ and EC_90_ values were derived from the regression equations showed in Supplementary Figures S1 and S2.

It is confirmed that *A. ochraceus* is more sensitive to eugenol than to menthol, while *A. niger* is more sensitive to menthol. In all treatments, *A. ochraceus* showed more susceptibility than *A. niger*. The combination of menthol and eugenol showed synergistic effect against the two fungi as their EC_50_ and EC_90_ values decreased when they were present as a combination (mix 1:1), compared to the case when each agent was used separately.

Previous research has indicated that major components of essential oils play a critical role in antimicrobial activities and they are less likely to change their biological properties than complex essential oils [9]. Thus, the antifungal activity of menthol and eugenol has been compared with that of the essential oils from which they are derived. Soković et al. [14] reported the effectiveness of *Mentha piperita* essential oil and their components as antifungal agents against several foodborne fungi. Mentha essential oil showed strong antifungal activity, but lower than that of pure menthol. Likewise, Prakash et al. [20] noted that eugenol, extracted as a major component of the betel medicinal plant (*Piper betle*), was more effective as a fungal growth inhibitor than the entire essential oil.

Additionally, the active dose of antifungals may vary in different studies depending on the experimental conditions. Marei et al. [21] performed a comparative study of antifungal activities of several monoterpenes on four plant pathogenic fungi including *A. niger*. Similar to our results, menthol at 200 µg/mL inhibited *A. niger* mycelial growth by 50% (in our study, 50% inhibition occurred at 300 µg/mL). However, Freire et al. [22] tested the antifungal activity of *M. piperita* essential oil, whose main component is menthol, and only managed to inhibit *A. ochraceus* and *A. niger* at concentrations of 1000 µg/mL (0.1%). In general, the higher the antifungal dose, the greater the observed effects. Abbaszadeh et al. [23] reported that increasing concentrations of menthol and eugenol resulted in progressive and significant growth reduction of some *Aspergillus* and *Cladosporium* fungi.

In the present study, menthol and eugenol combined as a binary mixture showed synergistic effects on fungal growth, since the activity of the combination was significantly greater than the sum of the activities of their individual compounds. In other studies, the combination of several antifungal agents has been shown to have synergistic effects. Ju et al. [24] reported that eugenol and citral showed synergistic properties for the inactivation of *A. niger* and *Penicillium roqueforti* in vitro and on bread. Their average minimum inhibitory concentration (MIC) decreased by 3.40-fold when used in combination (at a ratio of 1:1) compared to the case when each agent was used separately. The mixture of eugenol and citral inhibited the spore germination of both spoilage fungi by more than 95%, and their mycelial growth was almost completely inhibited.

Menthol, eugenol, and their combination exhibited notable antifungal efficacies on *A. ochraceus* and *A. niger* in the current study, resulting in smaller colony diameters. Safaei-Ghomi and Ahd [25] indicated that molecules containing a hydroxyl group (such as menthol) as well as a delocalized electron system in the phenolic ring structure (such as eugenol) showed antifungal activity. Regarding the mechanisms of action of the tested antifungals, several studies have shown that the main antimicrobial mechanism is through the increase in the permeability of cell membranes, leading to leakage of intracellular components and cell death [24]. Previously, Hua et al. [8] reported that eugenol causes morphological changes in the hyphae and conidiophores of *A. ochraceus* at concentrations from 250 µg/mL, as evidenced by scanning electron microscopy. They also reported that eugenol reduced ergosterol production in that fungus by 45–85% depending on the dose. Moreover, eugenol has been reported to inhibit the H+ ATPase system, leading to intracellular acidification and cell death [26].

During the present study, the antifungal activity of menthol, eugenol, and their combination was also carried out using the liquid dilution method to estimate the MIC and MFC values. The MIC values against *A. ochraceus* were 500 µg/mL (menthol), 400 µg/mL (eugenol), and 300 µg/mL (mix 1:1), while the MIC values for *A. niger* were 500 µg/mL (menthol), 600 µg/mL (eugenol), and 400 µg/mL (mix 1:1). For comparison, Hua et al. [8] reported that the MIC value for eugenol against *A. ochraceus* was 600 µg/mL (as compared to 400 µg/mL in present study), indicating that different strains may have somewhat different sensitivities to the same antifungal agent. Likewise, Ju et al. [24] reported that the MIC value for eugenol against *A. niger* was 250 µg/mL, a lower value compared to the 600 µg/mL in present study. In parallel with obtaining the MICs, the MFCs were determined with respect to *A. ochraceus* and *A. niger* at >600 µg/mL for menthol, eugenol, and the combination mixture. These results show that the activity of menthol and eugenol at the concentrations tested can be considered fungistatic (reversible). This is in agreement with Hua et al. [8], who reported that fungistatic concentrations of eugenol against *A. ochraceus* ranged from MIC (600 µg/mL) to 6.7 × MIC (4000 µg/mL), while from 4000 µg/mL, the inhibition was permanent.

**Figure 2:**
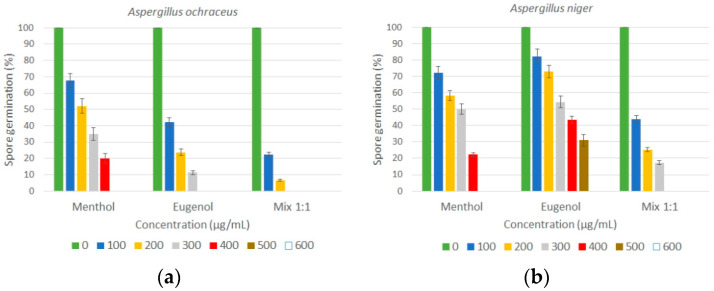
Effects of menthol, eugenol, and their combination (mix 1:1) on spore germination (%): (**a**) *A. ochraceus*; (**b**) *A. niger*. Results expressed as mean percentage ± standard deviation of three replicates.

On the other hand, the efficacies of menthol, eugenol, and their combination on the spore germination of *A. ochraceus* and *A. niger* are shown in Figure 2. The percentage of spore germination was significantly inhibited (*p* < 0.05) by the different concentrations of menthol, eugenol, and their combination after 24 h of incubation at 25 °C. The effects on fungal spores were dose-dependent, which was more evident as the concentration of the antifungals increased. Menthol and eugenol reduced the spore germination of *A. ochraceus* by 80.0% and 88.7% at 400 µg/mL and 300 µg/mL, respectively, while total inhibition was achieved beyond these concentrations. Again, the combination of menthol and eugenol (mix 1:1) caused most profound reduction in the spore germination of *A. ochraceus,* by almost 95% at 200 µg/mL, and by 100% from 300 µg/mL. For *A. niger*, menthol and eugenol at concentrations of 400 and 500 µg/mL reduced the percentage of spore germination by 77.7 and 69%, respectively. The combination showed a strong inhibitory effect, reducing spore germination to 17.33% at 300 µg/mL.

For comparison, Wang et al. [27] investigated the effect of nerol, a monoterpenoid alcohol found in many essential oils, and reported that the spore germination of *A. niger* reduced as nerol concentrations increased. The effects of menthol, eugenol, and their combination on spores may be due to the denaturation of enzymes involved in spore germination or interference with amino acids implicated in germination [28,29].

Results obtained with the in vivo fumigation with menthol and eugenol revealed their efficacy as a preservative for the control of fungal contamination in cereal grains (Table 2). Menthol and eugenol vapors showed noticeable antifungal activity when applied to maize, barley, and rice grains contaminated with *A. ochraceus* and *A. niger*. The treatments applied showed a greater than 50% protection of cereal grains from *A. ochraceus* and *A. niger* contamination. These results are in line with other studies in wheat grains [10] and couscous [30], where percent protection against *A. flavus* ranged from 52 to 77% and from 50 to 86%, respectively.

For *A. ochraceus*, the highest protective effect of menthol and eugenol was found in rice, where fungal counts in the control group (41 × 10^3^ cfu/g) dropped by 63.4% to 15 × 10^3^ cfu/g and by 70.7% to 12 × 10^3^ cfu/g, respectively. Likewise, menthol and eugenol showed greater protection against *A. niger* in rice, where fungal counts in the control group (15 × 10^3^ cfu/g) decreased by 60% to 6 × 10^3^ cfu/g and by 53.3% to 7 × 10^3^ cfu/g, respectively. Consistent with the results obtained in vitro, the fumigant effect of the mixture 1:1 was slightly higher (ranging from 62.8 to 75.6% protection) compared to the individual compounds. This indicates that the synergistic effect observed in vitro by direct contact is maintained when both agents are applied in vapor phase.

*Aspergillus* species reduce the quality and safety of cereals through the production of spoilage enzymes that cause discoloration and off-flavors, as well as through the synthesis of toxic secondary metabolites such as the mycotoxin ochratoxin. The application of vapors is an excellent technique for controlling food contamination because it leaves no residual components [13]. However, in no case were we able to obtain a total inhibition of fungal growth at the concentrations tested, which were equivalent to the MIC values with respect to the aerial volume of the container. Passone et al. [31] reported that contact exposure by clove essential oil, whose main component is eugenol, showed total fungal inhibition against *A. niger* growth at the level of 1500 µg/mL in vitro, while fumigation needed more than 3000 µg/mL. In previous studies, higher concentrations of plant essential oils are required in foods than in laboratory media, possibly because the lower water content of grains compared to culture media could obstruct the transport of antimicrobial molecules to the active site in the microbial cell [32].

Natural antifungals are more suitable than synthetic chemical fungicides from an environmental and safety point of view. However, high doses often negatively affect the sensory acceptability of the products, limiting their application [33]. Therefore, synergistic antifungal effects of a combination of different agents can be an effective way to solve this problem because the doses used are reduced. Additionally, by proving the synergistic effects of menthol and eugenol vapors in controlling fungal contamination, application methods can be designed to avoid direct contact between these agents and food. Thus, several researchers have developed recent approaches such as encapsulation, coating, and active packaging films for the use of antifungals in foods [34,35,36]. The results of the present study provide a scientific basis for the application of a combination of natural antifungals in the preservation of stored cereal products.

## 4. Conclusions

The most significant results of the present investigation were the efficacy of menthol, eugenol, and their mixture as inhibitors of the growth and spore germination of food-infesting fungi and their recommendation as plant-based food preservatives to improve the shelf life of stored food commodities. Using in vitro methods, menthol and eugenol showed remarkable antifungal effects (100% growth inhibition) on *A. ochraceus* and *A. niger* at concentrations from 400 to 600 µg/mL. The combination of both agents showed synergistic effects against both fungi, as complete inhibitory concentrations decreased to 300 to 400 µg/mL. The potential of using these compounds for grain preservation was further proved in the food system. Tested compounds and their combination exhibited pronounced efficacy in stored cereals, providing more than 50% protection against *A. ochraceus* and *A. niger* contamination in fumigated grains during 6 months of storage. These findings constitute a focus for the food industries on the potential application of menthol, eugenol, and their combination as food additives of plant origin.

## Figures and Tables

**Table 1:** Concentrations at which 50% (EC_50_) and 90% (EC_90_) of mycelial growth was inhibited in PDA after 7 days, expressed in µg/mL.

Compound	*A. ochraceus*		*A. niger*	
	EC_50_	EC_90_	EC_50_	EC_90_
Menthol	250	463	270	476
Eugenol	157	314	365	611
Mix 1:1	111	216	164	325

**Table 2:** Percent protection of cereal grains fumigated with menthol, eugenol, and their combination against *A. ochraceus* and *A. niger* after six months of storage.

Cereal Grain	Level (µg/mL Air)	*A. ochraceus* (cfu × 10^3^/g)			Level (µg/mL Air)	*A. niger* (cfu × 10^3^/g)		
		Control	Treated	% Protection		Control	Treated	% Protection
Maize								
Menthol	500	52	25	51.9	500	35	16	54.3
Eugenol	400	52	20	61.5	600	35	17	51.4
Mix 1:1	300	52	17	67.3	400	35	13	62.9
Barley								
Menthol	500	70	31	55.7	500	43	19	55.8
Eugenol	400	70	26	62.9	600	43	21	51.2
Mix 1:1	300	70	23	67.1	400	43	16	62.8
Rice								
Menthol	500	41	15	63.4	500	15	6	60.0
Eugenol	400	41	12	70.7	600	15	7	53.3
Mix 1:1	300	41	10	75.6	400	15	4	73.3

## Data Availability

The data presented in this study are available in the article.

## Supplementary Materials

The following supporting information can be downloaded at: https://www.mdpi.com/article/10.3390/foods12112108/s1, Figure S1: Linear regression of colony radial growth rate (mm) versus fungicide concentration (μg/mL) for *Aspergillus ochraceus* in PDA after 7 days; Figure S2: Linear regression of colony radial growth rate (mm) versus fungicide concentration (μg/mL) for *Aspergillus niger* in PDA after 7 days.
