# Supplementary material for: Antifungal Activity of Menthol, Eugenol and Their Combination against Aspergillus ochraceus and Aspergillus niger In Vitro and in Stored Cereals
Source: Foods. 2023 May 24;12(11):2108. doi: 10.3390/foods12112108 (PMC10252706; doi:10.3390/foods12112108)
Supplement: Supplementary file 1 [file foods-12-02108-s001.zip › foods-2389341-supplementary.pdf]

Supplementary Materials: Antifungal activity of menthol, eugenol and their combination against *Aspergillus ochraceus* and *Aspergillus niger in vitro* and in stored cereals

Yamina Ben Miri, Ahmed Nouasri, Marta Herrera, Djamel Djenane and Agustín Ariño

Foods

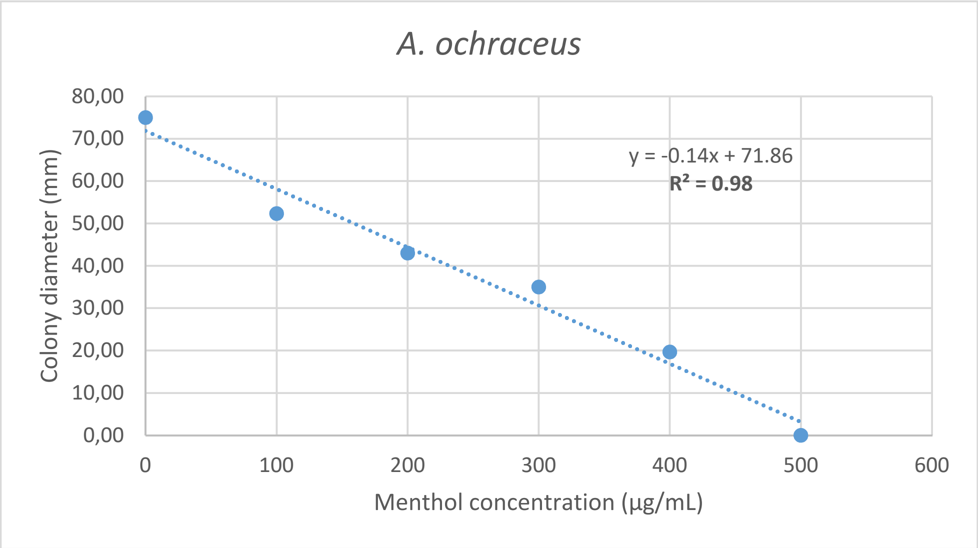

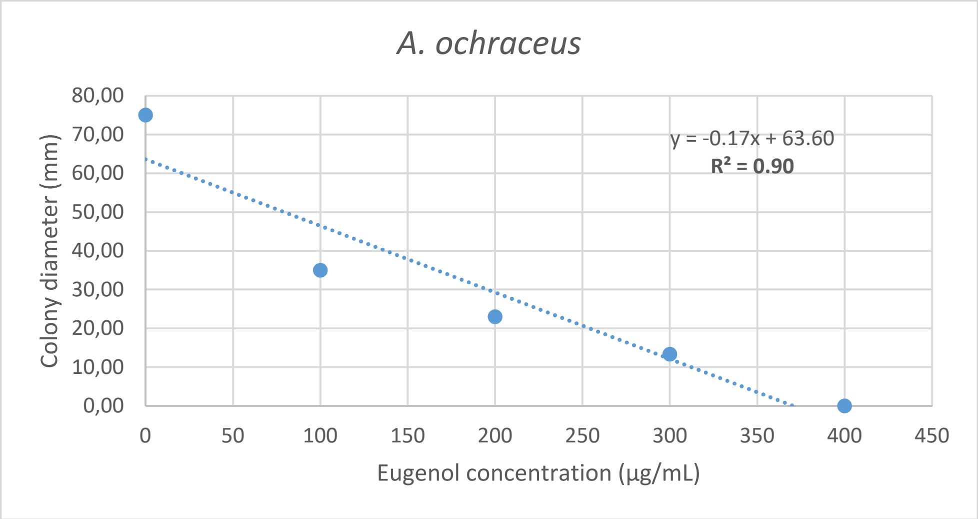

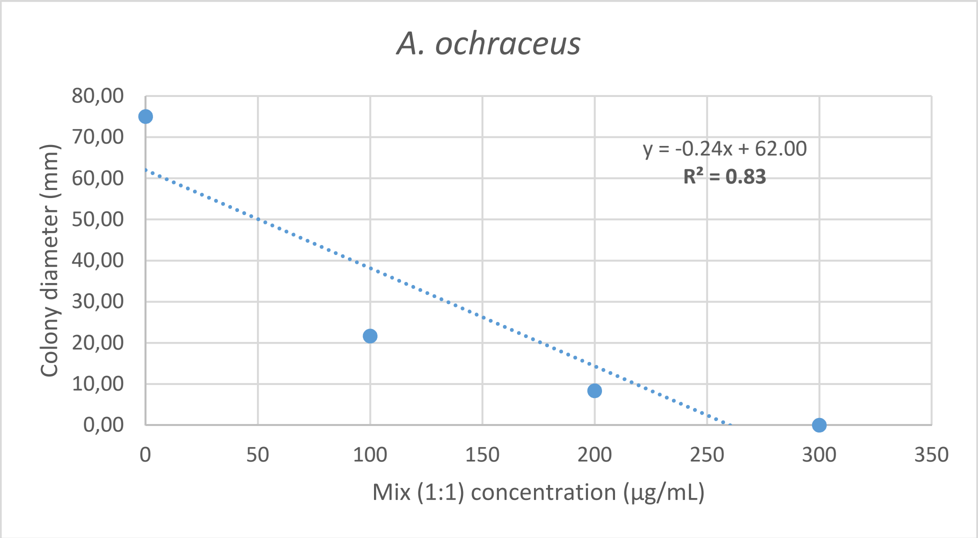

Figure S1. Linear regression of colony radial growth rate (mm) versus fungicide concentration (µg/mL) for *Aspergillus ochraceus* in PDA after 7 days

Supplementary Materials: Antifungal activity of menthol, eugenol and their combination against *Aspergillus ochraceus* and *Aspergillus niger in vitro* and in stored cereals

Yamina Ben Miri, Ahmed Nouasri, Marta Herrera, Djamel Djenane and Agustín Ariño

Foods

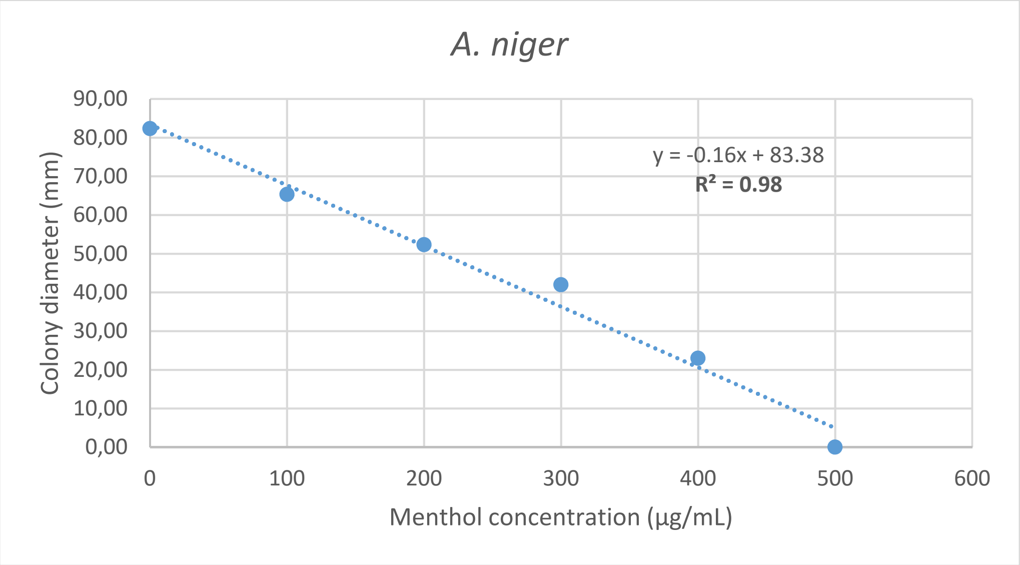

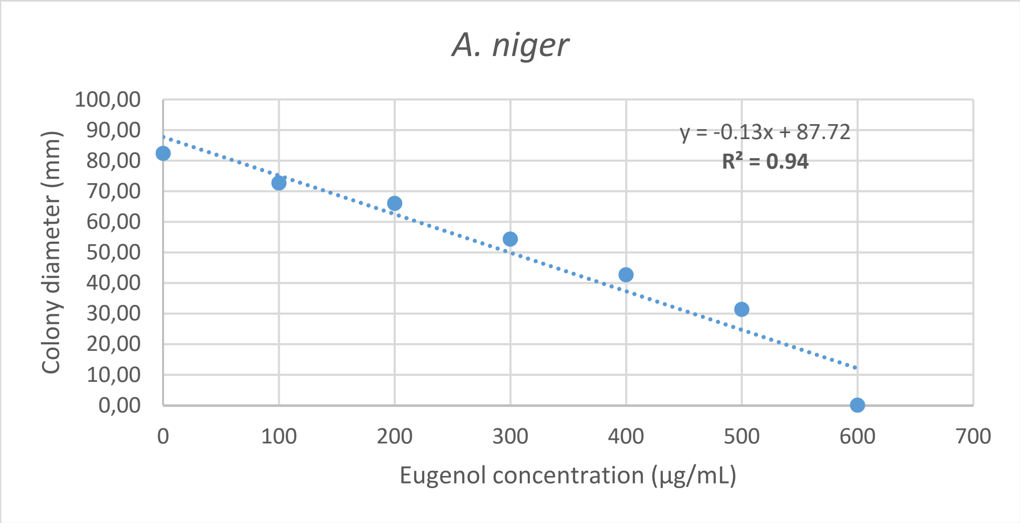

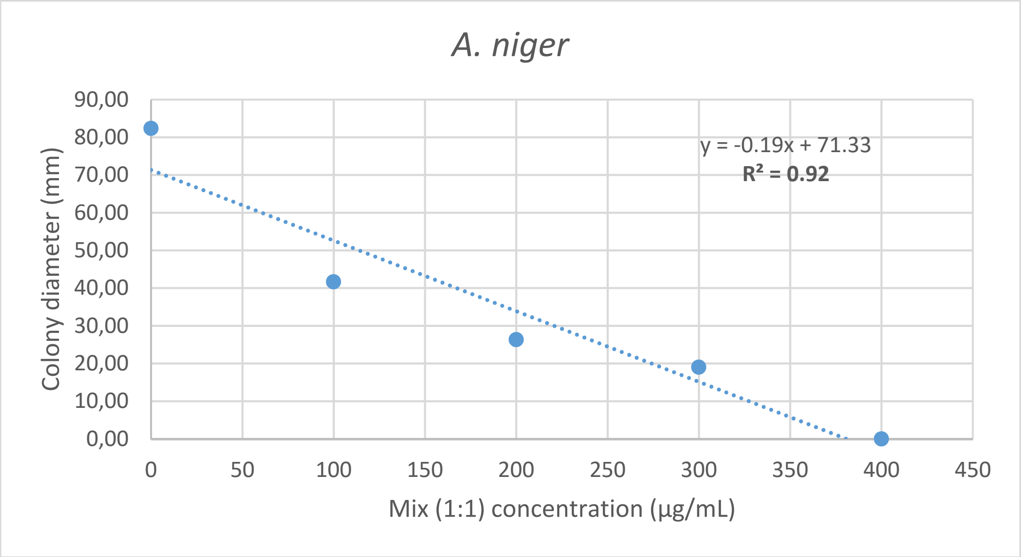

Figure S2. Linear regression of colony radial growth rate (mm) versus fungicide concentration (µg/mL) for *Aspergillus niger* in PDA after 7 days
